# Supplementary material for: Chronic kidney disease prediction using boosting techniques based on clinical parameters
Source: PLoS One. 2023 Dec 1;18(12):e0295234. doi: 10.1371/journal.pone.0295234 (PMC10691694; doi:10.1371/journal.pone.0295234)
Supplement: Attachment — Submitted filename: Response to Reviewers comments.docx [file pone.0295234.s001.docx]

**Response to Reviewers’ comments**

| **Reviewer** | **Reviewer’s concerns** | **Response/action taken** |
| --- | --- | --- |
| **Reviewer 1** | 1. The abstract must be re-written, focusing on the technical aspects of the proposed model, the main experimental results, and the metrics used in the evaluation. Briefly discuss how the proposed model is superior | We thank the reviewer for the suggestion. The abstract is written including the suggested points. |
|  | 2. Please make sure the abbreviations are properly used. For example, Area Under Curve-Receiving Operator Characteristic (AUC-ROC). | We apologise for the silly mistake. It is duly corrected. |
|  | 3. Additionally, method names should not be capitalized. Moreover, it is not the best practice to employ abbreviations in the abstract, they should be used when the term is introduced for the first time. | We acknowledge the reviewer’s concern regarding capitalisation. However, in the literature the normal convention to write the following terms are:  XGBoost, CatBoost, LightGBM, AdaBoost.  Furthermore, these terms are more popularly known by their abbreviated forms only. Writing the full forms for each of them would increase the word count of the abstract. Nevertheless, as suggested by the reviewer, the full forms of these terms are given in the text when they are used for first time. |
|  | 4. This paper is not a survey paper to include the references as "[30] [31] [32] [33] [34]." Please provide the significance of each of those studies and add a citation. | We thank the reviewer for the suggestion. The goal of the sentence is to establish the importance of ensemble learning in disease diagnosis and treatment. As suggested, we re- written the sentence by including sample research works that addressed different diseases using ensemble learning. |
|  | 5. Literature must be tremendously improvised for better idea on the field of study and state of art models, authors may consider some of the relevant studies like https://doi.org/10.1371/journal.pone.0271619 | We thank the reviewer for the suggestion. We included the suggested paper in the related work. We also improved the literature survey following the paper. A couple of recent papers are included in the related work. |
|  | 6. Boosting algorithms must be adequately explain, for better idea refer and include https://doi.org/10.3390/healthcare10010085 | We thank the reviewer for the suggestion. The boosting algorithms are elaborated with suitable mathematical equations. |
|  | 7. How are the feature weights calculated, what was the approach or the technique that is being used. | To calculate the feature importance, we used the wrapper method. This is included in the manuscript with suitable reference. |
|  | 8. Manuscript is having too many sub-sections, please minimize for better readability of the study. | We thank the reviewer for bringing our notice into this. There was a problem with the subsection 3.1 and Section 4 which should be included under Section 3. It is corrected duly.  We structured the manuscript into different subsections which allowed us to express the non-overlapping contents precisely and exclusively. This would help the readers to focus and understand the fragments of the experiment in a granular way. |
|  | 9. The section Data Standardization and Normalisation must be discussed as data pre-processing phase, that has to be in background section of the manuscript. | As suggested, the section Data Standardization and Normalisation is included in the Data Pre-processing section. |
|  | 10. What are the cases that are assumed as TP, TN, FP and FN, please explain them clearly, for better idea refer https://doi.org/10.3390/s21082852 | We thank the reviewer for the suggestion. Each prediction cases are stated with respect to Figure 7. |
|  | 11. Authors may present the loss functions for better comprehensibility of each of the models used in the proposed model. For better idea refer https://doi.org/10.3390/s21165386 | The misclassification rate of incorrect prediction is given in Table 4. |
|  | 12. Majority of the figures lack the clarity, they quality is fair but they must be explained in the text and the figures must be cited. | The figures are described and cited in the text. |
|  | 13. More comparative analysis with state-of-art models is desired. | As suggested by the reviewer, our work has been compared with a couple of papers in addition to the existing 6 papers, as shown in Table 5. |
|  | 14. By considering the current form of the conclusion section, it is hard to understand by PLos One Journal readers. It should be extended with new sentences about the necessity and contributions of the study by considering the authors' opinions about the experimental results derived from some other well-known objective evaluation values if it is possible | The necessity and contribution of this research work is included in the Conclusion section. |
|  | 15. English proofreading is strongly recommended for a better understanding of the study, and the quality of the figures must be tremendously improved. | The manuscript is proofread for possible grammatical and writing mistakes.  Regarding the figures, most of them are program generated and large in size. Accommodating in a smaller scale makes a couple of figures look unclear. However, they are perfectly readable by zooming. |
| **Reviewer 2** | 1. What are main aspects of novelty and advances in the described ideas? | In this paper, we proposed a novel CKD prediction model using ensemble learning. We used five different boosting algorithms to check the prediction performance of the model. Along with achieving better results (e.g., accuracy, precision, recall, etc.) and runtime we also assessed the contributions of all the attributes in the dataset that cause CKD. |
|  | 2. What are limitations of presented approaches? How the models work in different scenarios of operation? What are weak points of presented ideas? | We thank the reviewer for the suggestion. The limitations of the work in included in the Conclusion section. |
|  | 3. Related ideas: Deep neural network correlation learning mechanism for CT brain tumor detection, BiLSTM deep neural network model for imbalanced medical data of IoT systems, Deep learning for neurodegenerative disorder (2016 to 2022): A systematic review. | We thank the reviewer for the valuable suggestion. We’ve already started working on the deep learning based models for different disease predictions. |
|  | 4. Compare model to other in different operation and in different positioning of the input data. | We tried with different combinations of the data preprocessing, feature selection and hyperparameter tuning and came out with the best performing model. The model was tried with five boosting algorithms. Finally, the performance of the best working combination of the proposed model with AdaBoost is compared with a number of state-of-the-art findings. |
|  | 5. What are future trends in the development of this type systems? How the development would work in different configurations? What kind of transfer and network configurations are necessary for your model? | In future, more sophisticated disease prediction models will be prevalent in the medical diagnosis and treatment.  The proposed model can be used for other healthcare datasets that share the commonality of features.  The configuration setup will depend on the particular application requirement and the properties of the available dataset.  The future works of this work is mentioned in the Conclusion section. |
|  | 6. How to set optimal coefficients for these models? Did you test other configurations? How were these selected? | We tested the performance of the prediction model for combination of different coefficient values for all the tuneable parameters. Among them, the optimal value sets were selected, as shown in Table 3. |
|  | 7. Did you test the option to transform the knowledge before processing? | We carried out the background work to set up the base prediction model and tested it on different ensemble algorithms. Among them, in the given setup, AdaBoost performed best. |
|  | 8. There are no comparisons to other models so we are not able to see advances of your processing. | The proposed model is compared with several similar published works, as shown in Table 5. It can be observed that our model outperforms the other compared works. |
|  | 9. Your fig 1 is not much informative since there are no details on your model thus we are not able to repeat your experiment. | Fig. 1 shows only the overall flow of the paper. The details of each step are elaborately discussed in the manuscript. |
|  | 10. How do you understand T in your model? Is this a time of processing or number of iterations? | As mentioned in Section 6, it’s the runtime of the considered algorithms on the considered dataset. |
| **Reviewer 3** | 1. There are some typos and grammatical errors in Abstract. In addition, the abstract is not attractive. Some sentences in abstract should be modified to make it more attractive for readers. | We thank the reviewer for the suggestion. The Abstract is rewritten to make it more precise and attractive. |
|  | 2. In Introduction section, it is difficult to understand the novelty of the presented research work. In addition, some references are missing. | In this paper, we proposed a novel CKD prediction model using ensemble learning. We used five different boosting algorithms to check the prediction performance of the model. Along with achieving better results (e.g., accuracy, precision, recall, etc.) and runtime we also assessed the contributions of all the attributes in the dataset that cause CKD. |
|  | 3. In related work, the existing works about patient disease prediction should be discussed: ‘A smart healthcare monitoring system for heart disease prediction based on ensemble deep learning and feature fusion’, ‘Alzheimer’s disease progression detection model based on an early fusion of cost-effective multimodal data’, Automatic detection of Alzheimer’s disease progression: An efficient information fusion approach with heterogeneous ensemble classifiers’, and ‘An intelligent healthcare monitoring framework using wearable sensors and social networking data.’ | We thank the reviewer for suggesting the papers. We really appreciate the works presented in the suggested papers. The papers that are most closely related to our work are cited. |
|  | 4. The authors should properly select and check the subsection title. There are so many typos and (see section 3 research methodology. | We thank the reviewer for bringing our notice into this. There was a problem with the subsection 3.1 and Section 4 which should be included under Section 3. It is corrected duly. As suggested, a few sections/subsections are renamed. |
|  | 5. The number given to each section is not correct. | We apologise for the unintentional mistake. The numberings are corrected. |
|  | 6. Where are the other preprocessing steps? How is the data pre-processed? | The details of the data preprocessing are given in Section 4.4. The steps also pictorially shown in Fig. 1. |
|  | 7. What about feature selection? | We considered all the featured in the CKD dataset. We assessed the contribution of each feature in CKD. To calculate the feature importance, we used the wrapper method. This is discussed in the manuscript in Section 5.3.3 and also shown in Fig. 13. |
|  | 8. The results are not properly discussed. | The outcomes of the data preprocessing are presented in Section 4.2. The experimental results are discussed in Section 5.3. The performance of the proposed model is measured using several performance metrics such as accuracy, precision, recall, F1-score, support, AUC-ROC. |
|  | 9. Captions of the Figures not self-explanatory. The caption of figures should be self-explanatory, and clearly explaining the figure. Extend the description of the mentioned figures to make them self-explanatory. | We thank the reviewer for the suggestion. Most of the captions are rewritten for better understandability. |
|  | 10. Equation 2 should be more clearly discussed. | In the original manuscript, there was no Eq. 2. |
|  | 11. In conclusion section, the future work should be more deeply discussed. | As suggested by the reviewer, the Conclusion section is extended including the limitation of the work and future direction in this domain. |
|  | 12. The whole manuscript should be thoroughly revised in order to improve its English. | We thank the reviewer for the suggestion. The manuscript is thoroughly checked for English and grammatical mistakes. |
