# Supplementary material for: Chronic kidney disease prediction using boosting techniques based on clinical parameters
Source: PLoS One. 2023 Dec 1;18(12):e0295234. doi: 10.1371/journal.pone.0295234 (PMC10691694; doi:10.1371/journal.pone.0295234)
Supplement: Attachment — Submitted filename: Response to Reviewers comments (3rd review).docx [file pone.0295234.s003.docx]

**Response to Reviewers’ comments**

**Reviewer #1:** The authors have addressed all the recommendations of the reviewers in a reasonable manner, manuscript in the current from may be considered for the further phase of the editorial process.

-Captions of the Figures not self-explanatory. The caption of figures should be self-explanatory, and clearly explaining the figure. Extend the description of the mentioned figures to make them self-explanatory.

**Response:** We thank the reviewer for the suggestion. We renamed the figures and tables where required.

- The corresponding code may be enclosed to make the study evident.

**Response:** We agree with the reviewer’s suggestion. We plan to compile the code onto an open-access repository such as GitHub or our website soon.

**Reviewer #2:** Papers is presented in the same way as visible even after presented changes, thus i confirm my opinion from previous round

**Response:** In our communication with the editor, we will not need to respond this reviewer’s criticism because he/she continuously ignored what we revised our previous versions of manuscript in response to his/her comments.

**Reviewer #4:** Dear Authors, hereby is my initial comments:

1. The dataset used is a public dataset. You need to compare related works within the similar dataset instead of comparing with other datasets. To enhance your novelty.

**Response:** We appreciate the suggestion made by the reviewer. We compared our method with similar work that used the same dataset, i.e., the Chronic Kidney Dataset collected from the UCI machine learning repository. The comparison summary is given in Table 5. In the related work section, to discuss the state-of-the-art on chronic kidney disease prediction, we needed to explore all the recent work on the same topic.

2. The dataset is an imbalance dataset with ratio 150:250 for with/without CKD. In my opinion, you should make sure the dataset is imbalanced or else you need to use a proper performance measure for the imbalanced dataset. By having an imbalanced dataset, your results are biased results! See your results, CKD and non CKD have big gaps.

**Response:** We sincerely thank the reviewer for highlighting this omission. We’ve reworked with the dataset to make it balanced, as shown in Fig. 2. The whole experiment is conducted on this updated balanced dataset, ensuring that there are no biases in the outcome.

3. Did you select important features before doing hyperparameters tuning? How many features are discarded and how many features are retained? If so, the explanation of feature importance should come before hyperparameters tuning.

**Response:** We thank the reviewer for raising this query. In the revised manuscript, we eliminated the insignificant/non-contributing features from the dataset after finding the important features. Altogether nine attributes (‘ane’, ‘appet’, ‘ba’, ‘cad’, ‘pc’, ‘pcc’, ‘pe’, ‘su’, and ‘wc’) were discarded. We repeated the experiment with the modified dataset, the details of which are discussed in sections 5.2 to 5.5.

4. In standard ML procedure, important features are selected to be fed into the classifiers to improve the accuracy of the model.

**Response:** The reviewer has rightly stated that feeding the important features into the prediction model improves its performance. Complying with this suggestion, we repeated the experiment with the modified dataset (after eliminating the non-contributing features), the details of which is discussed in sections 5.2 to 5.5.

5. The wrapper method is used for feature importance. What type of wrapper method is used, Forward selection, Backward elimination, or Bi-directional elimination (Stepwise Selection)? Kindly explain.

**Response:** We thank the reviewer for mentioning this missed out point. We used forward selection as the wrapper method. It is now mentioned in the manuscript.

6. What are the initial parameters for each boosting algorithm before doing hyperparameters tuning? Table 3 is a found best parameters not hyperparameter tuning. Revise the table caption.

**Response:** We thank the reviewer for identifying the unintentional mistake. We tried with different values of the considered parameters. The best values found for each parameter are shown in Table 3. The caption of the table is changed as suggested.

7. Which dataset is used for hyperparameters tuning? 60% of the train set? What cross validation is used for this tuning?

**Response:** The hyperparameter tuning was done on the training set (i.e., 60%). We applied k-fold (k=6) cross-validation during the training phase itself. A separate subsection (5.4) is added in the manuscript to cover the validation.

8. What method is used for hyperparameters tuning? Grid search method for all algorithms? Kindly include the explanation of the grid search method.

**Response:** Besides the grid search method, we also tried with random search method but found that the grid search method provided the best results. Also, we preferred the grid search method because it is used in most of the literature for hyperparameter tuning in disease prediction. The further justification for using the grid search method is discussed in Section 5.3.

9. The accuracy results should have both for the train and test set. Figure 8 is a result for which set? The obtained confusion matrix in Figure 7 is for the test set?

**Response:** We thank the reviewer for the suggestion. We have added the training accuracy along with the testing accuracy in Fig. 8 (now Fig. 9). The confusion matrix shown in Fig. 7 (now Fig. 8) is of only the test set. Adding a confusion matrix of the training set would occupy another whole page. To keep the manuscript length standard, we decided not to add in the main text. If this reviewer still prefers this, we can do next time.

10. Kindly explain what happened in this stage c) Detect and replace the outliers. Why did you replace the outlier? The outlier is your real data.

**Response:** We thank the reviewer for this valuable point. Outliers are data points that considerably differ from the majority of the data in the dataset. They are generally resulted due to various errors. Outliers can significantly affect how well machine learning models function. They can skew the data distribution, making it non-normal or non-uniform. Therefore, it is important to deal with them. Removing or replacing outliers can make prediction models more robust and less susceptible to extreme values, allowing them to capture the underlying patterns more accurately.
